# Toward Better Food Security Using Concepts from Industry 5.0

**DOI:** 10.3390/s22218377

**Published:** 2022-11-01

**Authors:** Selvakumar Guruswamy, Milica Pojić, Jayashree Subramanian, Jasna Mastilović, Sohail Sarang, Arumugam Subbanagounder, Goran Stojanović, Varun Jeoti

**Affiliations:** 1 KPR Institute of Engineering and Technology, Coimbatore 641407, Tamil Nadu, India; 2 Institute of Food Technology, University of Novi Sad, 21000 Novi Sad, Serbia; 3 PSG College of Technology, Coimbatore 641004, Tamil Nadu, India; 4 BioSense Institute, University of Novi Sad, 21000 Novi Sad, Serbia; 5 Faculty of Technical Sciences, University of Novi Sad, 21000 Novi Sad, Serbia; 6 Department of Computer Science and Engineering, Nandha Engineering College, Erode 638052, Tamil Nadu, India

**Keywords:** food security, ICT, Industry 5.0, blockchain, IoE, digital twin

## Abstract

The rapid growth of the world population has increased the food demand as well as the need for assurance of food quality, safety, and sustainability. However, food security can easily be compromised by not only natural hazards but also changes in food preferences, political conflicts, and food frauds. In order to contribute to building a more sustainable food system—digitally visible and processes measurable—within this review, we summarized currently available evidence for various information and communication technologies (ICTs) that can be utilized to support collaborative actions, prevent fraudulent activities, and remotely perform real-time monitoring, which has become essential, especially during the COVID-19 pandemic. The Internet of Everything, 6G, blockchain, artificial intelligence, and digital twin are gaining significant attention in recent years in anticipation of leveraging the creativity of human experts in collaboration with efficient, intelligent, and accurate machines, but with limited consideration in the food supply chain. Therefore, this paper provided a thorough review of the food system by showing how various ICT tools can help sense and quantify the food system and highlighting the key enhancements that Industry 5.0 technologies can bring. The vulnerability of the food system can be effectively mitigated with the utilization of various ICTs depending on not only the nature and severity of crisis but also the specificity of the food supply chain. There are numerous ways of implementing these technologies, and they are continuously evolving.

## 1. Introduction

**Figure 1:**
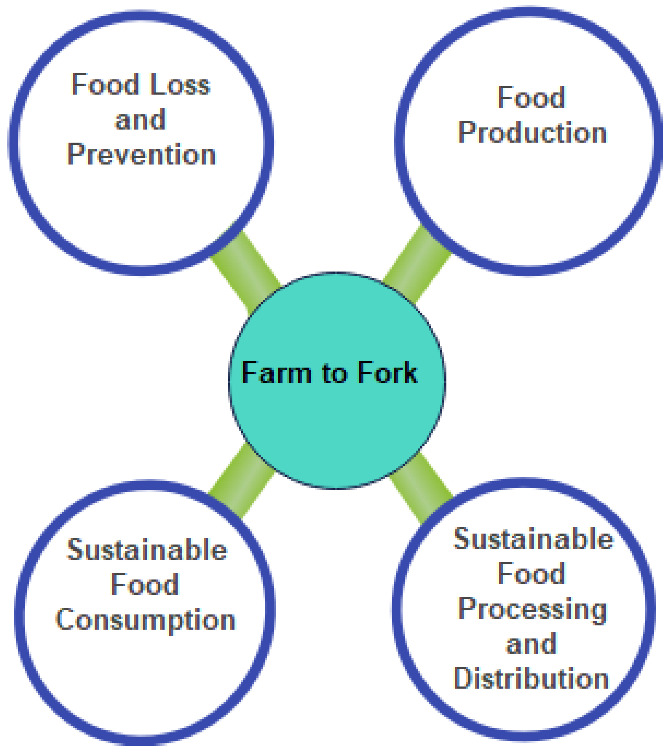
Farm-to-fork strategy.

Food security refers to a state of having access to adequate and safe food to meet the requirements of human beings [1]. According to [2] around 108 million people are facing extremely challenging global food security crisis, which is expected to rise as the human population is rapidly increasing and is predicted to exceed 10 billion by 2050 [3]. This further leads to an increase in the demand for food supply that will likely pose serious challenges to the food security with low food quality, food wastage, and lack of proper monitoring and testing in all processes involved in the food supply chain. A typical food system involves different stages and actors from agricultural production to retail distribution. Europe has developed a robust farm-to-fork (F2F) strategy to ensure a sustainable food chain from production to retail distribution while reducing food loss and mitigating climate impacts, as shown in Figure 1 [4].

However, the operations linked to the food system from F2F can be greatly affected by demographic and environmental changes such as climate-related natural hazards, including storms, droughts, and floods; pandemics; and war conflicts leading to significant food quality and economical losses [5]. Furthermore, these risks may cause insufficient food supply, economic pressure, shortage of labor, increase in poverty, and improper distribution of food [6]. In addition, the workforce and systems involved in inspecting and testing the food supply chains can also be greatly affected, as was the case with the COVID-19 pandemic measures, which raises serious concerns.

These factors have accelerated the need to build a more sustainable food system by bringing new innovative solutions to food system resilience while considering economic and environmental sustainability. For example, a multi-actor, multifaceted, and connected global strategy can be employed to support the development of sustainable food strategies and to ensure equitable food security [7,8]. In the literature, numerous existing works focus on food security, factors affecting it [9], challenges and new strategies to endure the food demands, and sustainability of food security [9,10]. Industry 4.0 aims to revolutionize the industrial processes from design to products by integrating information and communication technologies (ICTs) such as IoT, industrial IoT (IIOT), robots, cloud computing, big data analytics, artificial intelligence (AI), and machine learning (ML) [11]. These ICTs have introduced significant benefits in various applications [12]. Moreover, these technologies can be used for data collection, data transmission, processing and managing, and analysis in digital from. The latest developments in ICT tools drive to enable their applications in various fields including the food industry to address the challenges and restructure supply-chain activities to enhance the food security and integrity from F2F while reducing costs and waste [13,14]. These tools can further help in food processing, visualizing the factors affecting food quality, analyzing the food waste, and facilitating the decision-making process while considering the environment sustainability chain [15]. In addition to food quality, food integrity also plays a vital role in the supply chain that focuses on mitigating and preventing fraudulent activities such as mislabeling, adulteration, and counterfeiting by improving traceability and safeguarding in the food supply chain. Thus, ICT tools such as traceability software and radio frequency identification (RFID) have the potential to improve food traceability systems [16,17]. However, Industry 4.0 primarily focuses on maximizing productivity and achieving mass production using emerging technologies [18,19]. Its focus, therefore, is on digitalization of processes through pervasive sensing, IoT, AI, simulation, and digital twin. Robots are key to Industry 4.0 to achieve the stated efficiency in manufacturing, logistics, and retail.

Lately, with the emergence and evolution of Industry 5.0, there is an effort to use human expertise in collaboration with efficient, accurate, and intelligent machines to achieve resource-efficient and user-preferred manufacturing solutions [20]. Numerous promising technologies such as AI, big data analytics, digital twin, blockchain, cobots, 6G, internet of Everything (IOE), and edge or cloud computing are expected to continue to assist Industry 5.0 [20,21]. However, as emphasized earlier, the theme of Industry 5.0 is significantly different. It is more about technologies being sustainable and human-centric. The approach is increased collaboration between machines and humans—from robots to collaborative robots (cobots). Most technologies of Industry 4.0 continue to be of interest, yet the focus shifts from pure digitalization and machines to collaboration between humans and machines.

There are various existing works that discuss the potential applications of Industry 5.0 in different domains. For example, Maddikunta et al. [20] and Javaid et al. [22] provided a comprehensive survey on Industry 5.0 enabling technologies and their potential applications in smart healthcare, cloud manufacturing, production, COVID-19, and supply-chain management. Similarly, Mehdiabadi et al. [23] and Sharma and Arya [24] discussed the role of Industry 5.0 technologies in the banking sector and smart city applications, respectively. Akundi et al. [25] presented a thorough work on current research trends and analysis in Industry 5.0. Furthermore, Nahavandi [26] explained the key features and concerns of Industry 5.0 and its effect in manufacturing systems, whereas Jafari et al. [27] discussed the implications of Industry 5.0 for smart logistics. Despite this growing interest in using the technologies of Industry 5.0 for various application domains, most of the existing works, to the best of our knowledge, have not discussed their use in food security. This observation motivates us to provide a review on the use of Industry 5.0 technologies and their implementation in each process of the food supply chain with expected human-centric benefits by exploiting the potential and opportunities of these technologies in each component of the food system. For example, today, food logistics is still largely labor-intensive. Industry 4.0 would utilize robots in its version of smart food logistics, whereas Industry 5.0 would employ robots to collaborate with humans. Thus, the aim of this paper was to summarize the possible use of these technologies of Industry 5.0 in the food supply chain from agricultural production to distribution and retail. It highlighted the benefits of using these key technologies, such as big data analytics, AI, Internet of Everything (IoE), digital twin, blockchain, cobots, 6G, and edge or cloud computing, in a human-centric approach collaboratively so incorporated in the food system to achieve the sustainability, quality, integrity, and security in the supply chain. To the best of our knowledge, no other work has so far reviewed this domain.

The rest of the paper is organized as follows: Section 2 comprehensively describes food system and related factors such as security, safety and quality, traceability, integrity, food system resilience, and risks involved in food security. Industry 5.0 and its evolution are presented in Section 3 along with a brief description of its enabling technologies. Then, Section 4 focuses on digitalizing, tracking, and tracing the food supply chain. The expected key enhancements to the food supply chain by specific Industry 5.0 technologies are highlighted in Section 5. In the end, Section 6 concludes the paper.

## 2. Food System

**Figure 2:**
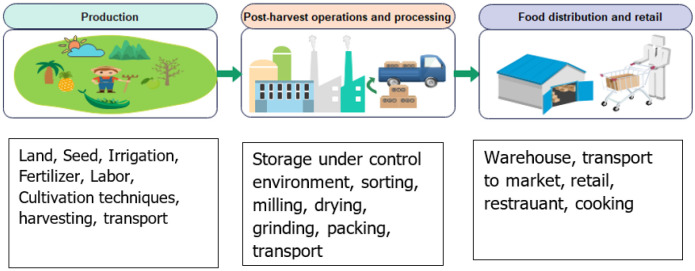
Typical food system.

According to the definition of the Food and Agriculture Organization, a food system is a collection of actors and their activities involved in the production, aggregation, processing, distribution, consumption, and disposal of food products as shown in Figure 2 [28]. This implies the complexity of food systems comprising different processes, value chains, actors, and interactions [29]. In general, the food value chain can be divided into two groups: the staple commodities characterized by high capital intensity (e.g., wheat, maize, corn, soybeans, and oil seeds) and the high-value commodities (fruits, vegetables, meat, and fishery) characterized by high labor intensity [30]. Rapid globalization significantly increased the international food trade (24% of produced food is internationally traded), whereas approximately 80% of the global population live in countries more or less dependent on food imports, which resulted in the increasingly connected food systems [31]. The specific patterns of trade connections between countries may intensify or diminish the transmission of production shocks to consumers [32].

Recent trends in consumer behavior highlight the increasing demands for local food, affecting the decentralization of food systems and shortening the food supply chains. The short food supply chains refer to a combination of food producers, distributors, and consumers in a smaller geographical area without a huge impact from the external world [33,34]. They already exist in the form of farmer’s markets, community-supported agriculture or solidarity, and purchase groups. Global food systems are characterized by extensive handling of commodities, ingredients, components, and finished food products involving many stakeholders before reaching the final consumer. To achieve the integrity of a food system based on quality, safety, and authenticity, the engagement of multidisciplinary actors is needed. To demonstrate the integrity of a food system, the entire food system should be controlled. Independent control by each food system player is not sufficient and cost-effective. Therefore, new diagnostic tools and the implementation of new information systems are required [35].

### 2.1. Food Security

Food security is defined as “a situation that exists when all people, at all times, have physical, social and economic access to sufficient, safe and nutritious food that meets their dietary needs and food preferences for an active and healthy life” [36]. To ensure food security, all processes within the food system must smoothly run. It is considered that the growing human population, shift in dietary patterns, limited natural resources, climate change, and environmental variability have already imposed challenges for ensuring food security. However, the crisis that sporadically occurs, such as wars and pandemics as recent cases, also has the potential to severely disrupt food security. To ensure food security, it is of utmost importance to understand the factors that contribute to the global food system’s ability to respond and adapt to such disruptions (i.e., resilience) [29,32].

### 2.2. Food Safety and Quality

Food safety refers to a system of proper food production, handling, processing, and trading that will have negligible to zero probability of contracting a foodborne disease. Food safety hazards can arise at any stage of the food system—from primary production to consumption. Food safety comprises (1) microbiological hazards associated with the presence of living microorganisms, which can cause food spoilage and possibly food poisoning; (2) chemical hazards associated with the presence of chemicals used in the agricultural and food processing to protect crops and livestock and enhance the quality of food (e.g., agri-chemicals, growth control hormones, feed conversion enhancers, and antibiotics); (3) toxic compounds (e.g., mycotoxins, biotoxins, and environmental contaminants); and (4) technological hazards associated with the utilization of technological advances with possible negative consequences (e.g., food irradiation and genetic modification of food) [37]. In a crisis, many constraints prevent running effective food control systems, which are mainly related to not only financial and human resource constraints but also the differences in food safety laws and regulations [38]. Nevertheless, in order to control food safety hazards and demonstrate compliance with food safety standards (HACCP, ISO 22000), entities in the food production chain have to monitor relevant safety indicators or regulators in critical points and keep transparent and reliable records regarding performed measurements. Therefore, the application of new methods to identify, monitor, and assess food safety hazards, coupled with data acquirement, storage, and presentation, is also required [39].

Food quality is the sum of all properties and assessable attributes of a food item, comprising sensory, suitability, and health value. Some of the attributes that make the quality of food are (1) *intrinsic* such as freshness, color, flavor, texture, taste, and nutritional value; whereas (2) the place of origin, the animal welfare, environmentally friendly production, and sustainable farming practices, *extrinsic* attributes of food quality as they are, are equally important [40]. Moreover, food quality has subjective, as perceived by consumers, and objective dimensions, as perceived by engineers and food technologists [41].

### 2.3. Food Authenticity and Traceability

Food authenticity is associated with the absence of any adulteration, especially related to the food composition, nature and varietal purity, geographical origin, and processing method. It is closely related with deception and food fraud attempts. In order to assure food product authenticity, the concept of traceability has been implemented. Traceability is defined as “the ability to follow the movement of a food through specified stages of production, processing and distribution” [42], and it is associated with the ability to link a final food product with its origin, ingredients, and processing. Traceability is of utmost importance for the global sourcing, centralized food manufacturing, and global distribution [43]. Nowadays, traceability is considered a new dimension of food quality [44]. The concept of food traceability heavily relies on the speed of the response, real-time detection, and the reliability of information across the food system, which has been significantly improved by the implementation of automated ICT-enabled and empowered systems representing a shift from manually operated and paper-based traceability [35]. Pizzuti and Mirabelli [45] proposed the global track and trace system (GTTS) and highlighted its potential to guarantee the origin and quality of food, assure the compliance with regulations, provide the consumer with additional information not generally given on the product’s label, and manage the entire products’ lifecycle. Moreover, the system enables the identification of noncompliances, avoidance of food fraud, reduction of off-season sales, and certification of the total quality of the product. Pearson et al. [46] highlighted the potential of distributed ledger technologies (DLTs) in general, and blockchain, to assist with food traceability by securely linking the entire food supply chain from the producer to end user. In such circumstances, if food safety or quality issue ever arises, the instant and accurate traceability to the origin and history of any particular food item will be possible.

### 2.4. Integrity of Food System

Studies that examined population dynamics, food production, and trade reported the increasing vulnerability of the global food system [32]. Vulnerability of the food system to either sudden or long-term shocks is due to its exposure to multiple internal and/or external causes. External causes of food system disruption are war conflicts, terrorist attacks, natural catastrophes (e.g., earthquakes), and extreme weather conditions (e.g., floods, droughts, cyclonic storms, hurricanes, and tsunamis) with high incidence in recent years due to climate change. The external disruption of food systems can be caused by not only the outbreaks of pests but also chronic poverty, economic crises, or political instability [29,47]. The internal causes of food system crisis are associated with human errors and the emergence of new biological and/or chemical agents, as was the case with the epidemic of cholera in Latin America in the early 1990s; bovine spongiform encephalopathy (BSE) in the UK in 1996; dioxin in Belgium in 1999; worldwide acrylamide and semicarbazide crisis in 2002 and 2003, respectively; melamine in China in 2008; and Escherichia coli O104:H4 in Germany in 2011 [48]. These shocks can significantly disrupt food supply and security, livelihoods, and human well-being either at the local or international level [49].

The possible scenarios in crisis are connected either with scarcity of food as happened in 1978 and 2007–2008 due to drought in grain-producing countries, or with surplus of food, which cannot be marketed. The difficult access to the market resulted in the accumulation of food, especially fresh produce at farms, causing food losses [50].

The onset of crisis, to a great extent, depends not only on the reaction of the public, industry, and governments but also on the geographic location of occurrence and the perception of risk, culture, and societal values [48]. The lack of information on market conditions (production, stocks, consumption, trade, and prices) and uncoordinated policy interventions by countries in 2007–2008 caused the disruptions of food systems and food price rise. Moreover, in the crisis, the focus is shifted to resolving the crisis, whereas the human resources that are normally engaged in the inspection of quality, safety, sustainability, authenticity, and other attributes cannot function to an adequate extent. In that case, data from private databases and papers could be made transparent with the application of adequate IT solutions to reduce frauds, corruptions, and errors [51,52].

The recent outbreak of the COVID-19 virus around the globe has severely disrupted the access to food for a vast number of people, not necessarily due to the limited food availability but rather due to the reduced food access caused by the altered regimes of food supply, changed working hours, restricted movement of citizens, and hoarding of groceries caused by panic buying [53]. Marusak et al. [34] showed the adaptability and responsiveness of regionalized food supply chains within the US food system to changes in demand and delivery during the COVID-19 crisis mainly based on the willingness of farmers and food distributors to adopt newly imposed strategies. 

The impact of crisis situations on the food supply chain is dependent on the type of the food supply chain. So far, it seems that the recent COVID-19-related crisis less impacted staples supply due to the restrictions on movement, although the potential threat can arise from the limited transportation across cities, provinces, regions, and countries, as well as export restrictions. This indicates that countries more dependent on imported food are more vulnerable to the crisis and more exposed to the threat of rising food prices, which consequently would lead to the reduction of purchasing power of citizens [50]. On the other hand, the supply chain of high-value commodities, as being high labor intensive, has been more affected.

Recent crises, including the coronavirus outbreak and the ongoing war in Ukraine, have shaken up all stakeholders in food value chains—from farmers to consumers—imposing the need to adapt to the new situation in a very short time. It is expected that the prolongation of the COVID-19 pandemic crisis and war in Ukraine will seriously affect food systems, especially due to closure of ports and logistic hurdles [50]. Apart from food security, the crisis situation may impose new challenges to ensuring proper food quality, safety, and authenticity.

### 2.5. Food System Resilience

Food system resilience can be described as its ability to respond and adapt to disruptions while maintaining its functioning [54]. Seekell et al. [32] considered three dimensions of food system resilience: (1) the ability to access food, (2) biophysical capacity to increase food production, and (3) diversity of domestic food production. Moreover, they emphasized the problems in the application of quantitative methods for tracking changes in food system resilience. Therefore, a development of an index-based analysis of the capacity of countries to handle shocks in the food system was suggested. Access to food is associated with prices and income and the ability to respond to the price spikes, crop failures, or loss of assets. Moreover, it is associated with the level of education and the investments in infrastructure [32].

### 2.6. Risks Involved in Food Security

Conventionally, the food system involves different actors and activities such as growing, processing, distributing, consuming, and disposing of food [55,56]. Natural hazards such as droughts, floods, disease, and wildfires pose serious risks to the agri-food sector. The impacts of such events lead to imminent loss to agriculture, livestock, and forestry, causing a decrease in crop yield and disruption in food production. For instance, some countries have limited access to water, which negatively affects food production. On the other hand, floods cause widespread damage to the physical infrastructure including farms and crops, resulting in reduced agricultural yields and a negatively affected food supply chain. Furthermore, insect pests, pathogens, ticks, and locust outbreaks are also potential risks to food security [57]. Moreover, the food system, especially the production process, heavily relies on pesticides, chemical fertilizers, and antibiotics, which degrade the land, surrounding water body, and environment, leading to high losses in biodiversity.

Recently, the COVID-19 pandemic has created a significant impact leading to high risks to global food security. The outbreak has weakened the food system operation from food production, transportation, and distribution to consumption. The COVID-19 restrictions have led to labor shortage and travel bans, resulting in the uncertainty in meeting the food supply and demand and the increase in food prices. Furthermore, some food products including fruits and vegetables have low shelf life. This was significantly affected by COVID-19-related quarantine measures for suppliers and caused massive food waste and financial losses to the farmers as well as stakeholders.

Concerns about food security arise even further in a war situation due to the suspension of commercial operations, destruction of infrastructure, and disruption of business activities leading to unemployment and halting food exports. Thus, these factors severely affect the food supply chain and cause long-term food insecurity. Additionally, scarcity of food forces the displaced population to access food at a high cost. Moreover, actors involved in the food system from production to consumption also get affected due to the destruction of agricultural farms and storage facilities, and the migration of the workforce, hampering the food supply chain. Furthermore, the war also leads to high inflation because of sanctions, blocking the import and export transactions. For instance, the ongoing conflict between Russia and Ukraine is a serious challenge to food security as both countries are major exporters of wheat. Last year, their share of global exports of wheat increased to 18% and 10%, respectively.

These risks strongly affect food security, which is a serious concern. Therefore, there is a need to transform the traditional food system into a more sustainable solution from agricultural production to consumption. The emerging technologies of Industry 5.0 can help to transform traditional food systems toward digitalization to ensure food security, consumer demands, and proper resource management and support in the decision-making process.

## 3. Industry 5.0 and Its Evolution

**Figure 3:**
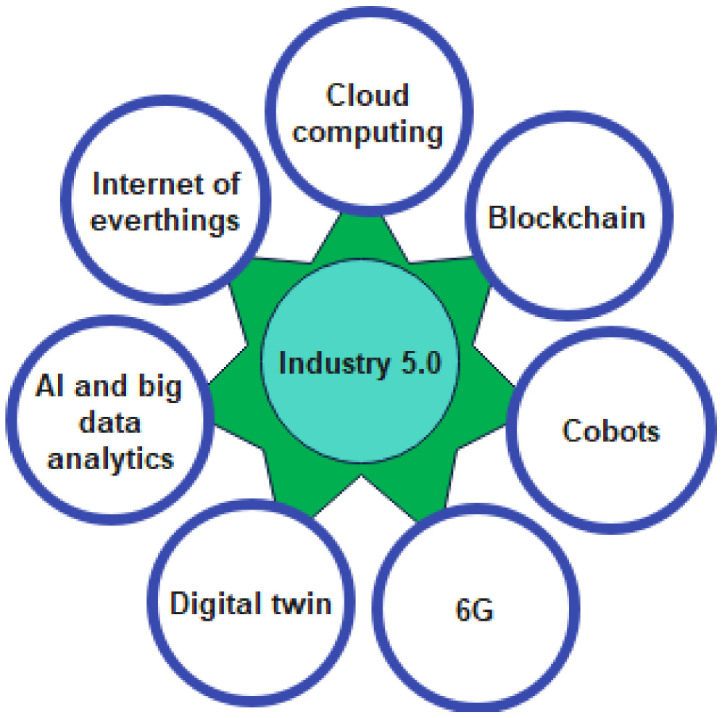
Enabling technologies of Industry 5.0.

Industry 5.0 is targeting the next industrial evolution that aims in advancing the collaboration between human experts and efficient and intelligent machines to achieve resource-efficient and user-preferred manufacturing solutions [20]. The main technology drivers of Industry 5.0 are artificial intelligence (AI), big data analytics, digital twin (DT), blockchain, cobots, sixth-generation communication technology (6G), Internet of Everything (IOE), and edge or cloud computing, as shown in Figure 3. These latest technological trends in collaboration with highly trained and equipped specialists aim to enhance manufacturing efficiency, quality of the production, and timely response to the users according to their needs, named mass personalization. The key enabling technologies of Industry 5.0 are briefly discussed hereafter.

### 3.1. AI and Big Data Analytics

Big data has gained major focus in both the industry and academia [58]. It can be defined as a large and diverse set of data gathered from different sources, e.g., sensors. The big data is next processed for data analysis to extract useful trends and information. There are many data analysis approaches available, such as AI, machine learning (ML), data mining and data fusion, and others that can be utilized in the food system [59]. These data analytics approaches can help in the better understanding of system behavior and can optimize system performance. In addition, analysis of data collected from social platforms, such as Facebook and Twitter, can further help in product customization and checking consumer satisfaction. Furthermore, big data analytics can be incorporated for prediction, which will lead to smart decisions.

### 3.2. Digital Twins

The evolution of AI and advanced data-processing techniques leads to adopting the digital twin, which is the virtual representation of a device or process or system including both its elements and dimensions and how it works throughout its lifecycle in a virtual space. The digital twin was first introduced by NASA in 2002 and used for monitoring the spacecraft’s behavior. Since then, the concept has significantly matured with the development of new technologies that can bring the industrial process to new levels of productivity and sustainability. DT enables connections between physical and virtual spaces, estimates product quality, supports in identifying failures and their causes, reduces costs and downtimes for maintenance, and optimizes the food supply and decision-making processes [60,61].

### 3.3. Cloud Computing

The IoT applications collect a large quantity of data, which requires proper data processing to store and extract the necessary information. Utilizing cloud-based technologies such as Microsoft Azure enables users to directly securely access the data from the cloud while eliminating manual measurements. These cloud computing (CC) services have achieved much attention in an industrial process to store the data gathered using sensors associated with each process from production to distribution and consumption and transfer to the cloud for further analysis [62]. In a food system, data related to food processing and traceability systems can be stored on a server before processing for further analysis. The available data can be utilized to predict trends and quality parameters to enhance the supply management and decision-making process.

### 3.4. Internet of Everything

The IoE extends the concept of the IoT by interconnecting humans with processes, data, and things and supports the integration of AI with smart devices, sensors, and actuators to support a wide range of applications. It introduces new opportunities and functionalities in Industry 5.0 and can help to improve customer experience and minimize cost. It covers a wide range of Internet-based concepts such as the Internet of People (IoP) and industrial Internet (II) [63]. The IoE can extract and analyze data gathered from different sources such as sensors, actuators, drones, and robotic devices for human actors that make the supply-chain process more intelligent and productive. However, appropriate measures must be taken at the initial phase of design and implementation. In the supply chain, the IoE may help to minimize waste and optimize the production process using information shared between humans and sensors [20].

### 3.5. Blockchain

Data security and transparency is a very important factor for the success of the Industry 5.0 ecosystem. The blockchain technology has been developed and so introduced that it offers a higher level of data security using the concept of decentralized and distributed ledger systems [64]. It stores information, e.g., transactions, and makes it available to all entities. It uses encryption algorithms as well as smart contracts that help automate the agreement process between different entities. In the blockchain, it is almost impossible to manipulate the recorded data since blockchain enhances data authentication between two parties or in a process. As a result, it has gained widespread attention in the supply-chain domain to improve data transparency and enhance trust among participants involved in the food system [65].

### 3.6. Cobots

Massive technological developments of Industry 5.0 are seeking to use human capabilities in collaboration with robots, i.e., collaborative robots, called cobots, for different applications including manufacturing processes [26]. These cobots are equipped with smart sensors, which are highly responsive and can detect any unwanted event that occurs in their path. In addition, they offer a significant contribution to Industry 5.0 by providing user-preferred and resource-efficient solutions and fast product delivery to customers [20]. Furthermore, it facilitates workers and helps to enhance the productivity and robustness of the supply-chain process.

### 3.7. 6G

The rapid growth in potential applications of existing 5G communication technology is driving new technological advancements to meet the resulting bandwidth and infrastructure requirements [66]. The new 6G technology, which is expected to be witnessed in the next ten years, can help to meet connection speed, reliability, and coverage requirements. 6G in collaboration with other technologies can offer substantial value-added services to Industry 5.0 and can support high-quality services in the supply-chain process. Moreover, it is expected that 6G may lead the world toward a more data-driven, intelligent, and digitalized environment that can support a wide range of industrial functions with ultrahigh reliability, data rate and energy efficiency, and ultralow latency [67].

## 4. Digitalizing, Tracking, and Tracing Food Supply Chain

### 4.1. Agricultural Production Process

**Figure 4:**
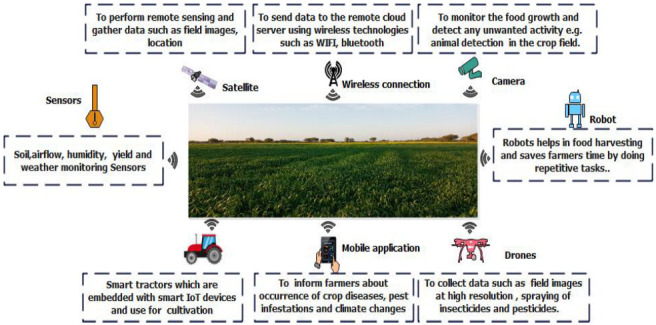
ICT tools used in agricultural production process.

The agricultural production process involves different production practices that depend on the food demand and overall impacts. The most common production practices are crop cultivation, livestock, fisheries, and aquaculture. In this process, different ICT tools such as sensors, drones, robots, smart cameras, and satellites are used to monitor and gather data from the food production field. The description of traditional ICT tools used in the agricultural production process is provided in Figure 4.

Sensors represent one of the data acquisition technologies that, when incorporated into the food-supply-chain infrastructure, enable data collection, processing, storing, and analysis. A vast number of research articles have been published in the recent past that studied the use of sensors to monitor parameters such as temperature, humidity, soil conditions, and location and obtain actionable insights. The development and implementation of sensors within food systems can enable better detection of food degradation markers and, thus, prevent and reduce food losses. Imaging sensors aboard satellites and/or drones or on physical structures on land represent one of the data acquisition technologies that enable the capturing of images for subsequent image analysis by different algorithms [68]. By their implementation in precision agriculture, most manual methods for direct measurement of phenotypic data (e.g., biomass, leaf area index, chlorophyll content, and plant tissue nitrogen content), which required intensive field collection of samples and destructive and time-consuming measurements, have been replaced by remote and nondestructive methods [69]. They can be mounted on tractors, robots, and drones. However, the listed imaging sensors due to their high cost and weight are often inadequate for mounting to drones, while the resulting images are difficult to process.

Robotics is an area in which mechanical, electrical, electronics, and computer science innovations come together to develop machines that can perform a specific activity to replace manual effort. While most of the robotics applications are still in the prototype phase, significant success in a few important areas has been achieved. Moreover, the agriculture field is an extremely dynamic environment, and the robots are expected to operate with more intelligence and precision [70]. Robotics technology is assisted with AI, ML, and deep-learning techniques to make the decision-making process less faulty, but, still, there are a lot of challenges that need to be overcome to improve the practicability and accuracy of the automatic recognition models [71].

On the other hand, common digital camera-based visible light sensors, although easy to operate and inexpensive, are inadequate due to their lack of red-edge and near-infrared bands, which have proven useful for more in-depth research and vegetation monitoring applications [72]. The utilization of unmanned aerial vehicles (drones) for agricultural purposes is becoming more and more important, and it is predicted that agriculture will become the second-largest user sector in the next five years after the military sector. Their utilization is mainly based on computer vision technology and capturing low-altitude high-precision images above the crop field and image processing to extract valuable data about the field and crop conditions (e.g., moisture status, soil properties and tillage mapping, disease and weed detection, etc.) [73]. Satellites are also used in agriculture fields for different parameters such as soil, drought, snow cover, and crop growth. These different sources deployed on the production location collect data and send them to the gateway and cloud using wireless communication technologies such as WiFi, Bluetooth, LoRa, and 4G/5G. However, data collected from the production field may face security attacks such as eavesdropping, disruptions that can alter the information [74]. Thus, it is also necessary to ensure data reliability and establish trust in the food system. In addition, devices deployed on the production field may be exposed to the surrounding environment that can also affect the data transmission. Furthermore, massive technological developments such as IoE enable smart sensors that are highly responsive and gather a large amount of data, resulting in increasing bandwidth, data storage, and infrastructure requirements. Thus, the rapid growth in ICTs such as Industry 5.0 technologies, e.g., IoE, blockchain, and 6G, can help the food production process by allowing sensors, drones, satellites, and robots to collect data and transmit and store them on a server timely and securely. Then, AI and DT utilize the stored data in a more intelligent way for prediction and utilize the findings in a virtual space to remotely optimize the production.

### 4.2. Postharvest Operations and Food Processing

This process transforms the primary production output such as crops, fruit, livestock, and seafood into secondary products that include meat, oil, sugar, coffee, grains, drinks, baking products, cereal, etc. Food processing involves food cleaning, packing, and storing that follows a set of standards relating to food security and hygiene and legal standards related to food safety (HACCP, ISO 22000). Postharvest operations and food processing involve different ICT tools. For example, sensors are used for monitoring incoming food production outputs and for identifying food quality, as well as for monitoring relevant indicators related to food safety, such as temperature, moisture, composition, presence of microorganisms, etc. Traditionally, RFID technology and barcodes are used for tracking and tracing, which are also a precondition of food safety management. The RFID tag is always attached to the product that can communicate (via radio wave) with the reader. Then the reader provides the information about the food product to the operator that can be accessed using a computer or mobile application. RFID tags are easy to use and do not need line of sight. They provide a good data rate and memory size. However, these tags rely on the reader for data gathering, and they cannot initiate communication. In addition, they are costly and have limited capabilities such as range. The barcodes are also gaining significant attention to digitally store the data. A barcode consists of a 12-digit numerical string that is assigned to each product, called the Universal Product Code (UPC) [75]. The food supplier maintains the record of the UPC associated with food products that describes the type, quantity, manufacturer, and origin of the food product. Barcodes are economical and provide good traceability, but they require line-of-sight access and do not support simultaneous multiple product scanning. In case of damage, barcodes become unreadable.

**Figure 5:**
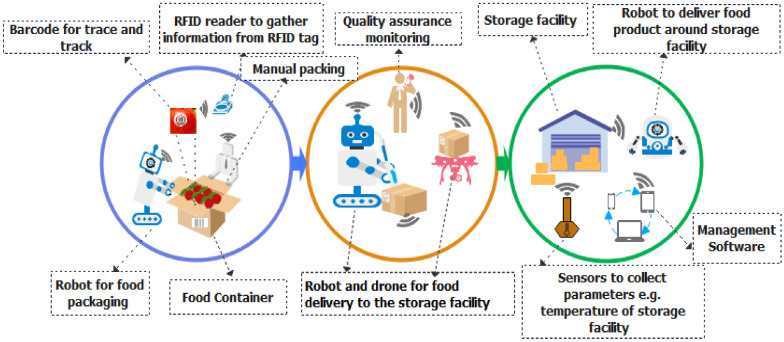
Postharvest operations and food processing.

The data gathered from sensors are sent to a remote cloud server using wireless communication technologies such as WiFi and Bluetooth, as given in Figure 5. The gathered data at the server will be used to monitor the progress of the food processor and for data analysis. The utilization of AI and ML algorithms will allow management to take better decisions to reduce operational costs and improve productivity. The utilization of computer vision and artificial intelligence in food processing industry has enormous potential, which has recently been critically reviewed by Kakani et al. [76]. For example, computer vision technology, which provides the automatic visual inspection and measurement using digital cameras and image analysis techniques, has found its wide application in food industry mainly due to its ability to contribute to the product quality improvement, throughput increase, waste reduction, and prevention of the overuse of ingredients [77].

In food processing, different computer vision systems are available differing in complexity. The simplest vision systems comprise self-contained cameras with built-in image triggering, lighting, and embedded image analysis capabilities. Application of different computer vision techniques has been demonstrated within different branches of the food industry and in different processes in postharvest and processing operations.

Although the global food industry is one of the biggest manufacturing sectors, and there are evident benefits that robotics technology can provide in food processing, the rate of their uptake is still slow in comparison with other manufacturing industries [78,79]. Robots can be defined as highly automated mechanical manipulators controlled by computers [80]. Bader and Rahimifard [78] reported the benefits of the utilization of industrial robot methodology in food processing, such as cost savings, improved productivity and product quality, and replacement of human operators in unsafe and unappealing operations. As they provide the improvement of maintaining the hygiene standards, their utilization during the pandemic crisis is highly relevant. Khan et al. [80] gave a detailed analysis of robot requirements in the food industry: kinematics, dynamics, hygiene, productivity, high precision, reliability, repeatability, safety, cost, and ease of operation and maintenance. In relation to the application, robots in the food industry can be classified as follows: pick-and-place robots, palletizing robots, packaging and labeling robots, product inspection and testing robots, and cooking and serving robots. To increase the uptake of robotics technology in food processing, challenges related to food characteristics and their sensory properties (such as fragility, nonrigidity, irregular shape, and stickiness), hygiene requirements (must be made from nontoxic, noncorrosive, and easily washable materials, free of any crevices), economic and social barriers (such as technophobia and fears of redundancy), and shortages of skilled operators [78] must be met. Duong et al. [79] emphasized the fact that the utilization of collaborative robots (so-called cobots) that can work side by side with human labor has not been sufficiently explored. It is speculated that the integration of cobots can transform the food processing industry, where safe collaboration with human workers can increase the utilization of highly skilled workers and reduce health risks. Certain robotics applications do not require direct contact with robots, whereby human workers are required for monitoring and remote control of the production lines. The utilization of robots is highly important in operations associated with potential hazards [79]. Guiochet et al. [81] emphasized safety as the main limitation for their deployment in real life. In this context, safety is related to users, the environment, and the robot itself. Due to the human–robot physical interaction, Guiochet et al. [81] emphasized the need for an adaptation or revision of the safety techniques. Moreover, the challenges related to the high costs of robotics systems should be overcome to allow their wider adoption. The robotics applications are based on the use of sensors, and although their price has recently declined, they are still considered more qualitative than quantitative. Nevertheless, in a traditional postharvesting and food processing approach, trace-and-track data related to the food product are directly sent to the centralized system. In case of failure, it may disrupt all food operations. Furthermore, it significantly depends on the surrounding parameters such as ventilation, temperature, relative humidity, gases, and location that can affect the quality and shelf life of food products [82]. Sensors that collect these parameters may also be exposed to the surrounding. For example, change in temperature can disturb radio link quality and may lead to packet loss. In addition, some sensors, e.g., alert sensors, may need to follow strict delay limits and require reliable and timely transmission. ICT tools such as sensors are usually equipped with batteries, which have limited lifetime and need continuous monitoring to avoid any disruption in operation. Thus, it is necessary to incorporate the latest technologies of Industry 5.0 to overcome these challenges in postharvesting and food processing. For example, stand-alone energy-harvesting technologies or, alternatively, rechargeable batteries assisted with energy harvesting can be used to avoid the limited lifetime issue of batteries. Similarly, blockchain can be used that offers a decentralized management platform that can help in avoiding the risk of single-point failure faced by the traditional system resulting in a consensus that blockchain can improve the transparency necessary for food safety management in food supply chains according to the demands of contemporary food safety standards [83]. 5G and 6G infrastructure technologies can help to achieve fast and reliable data transmission to remote servers for data analytics. DT enables creating a replica of physical elements in the virtual space and utilizes the stored data in a more intelligent way to remotely enhance the supply-chain process.

### 4.3. Food Distribution and Retail

**Figure 6:**
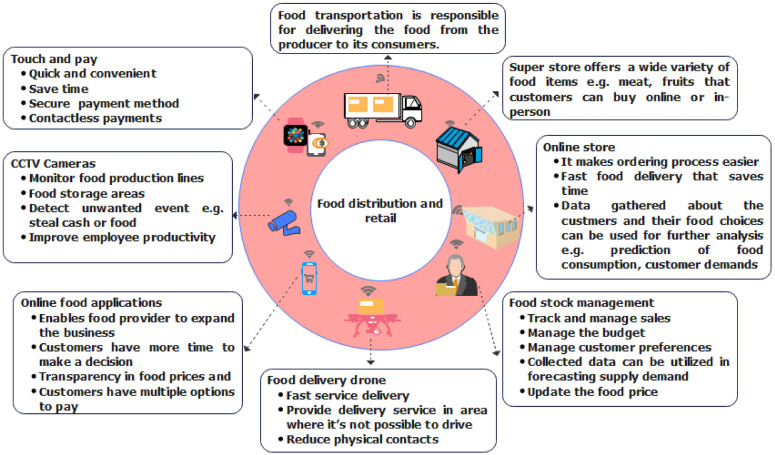
Food distribution and retail.

Food distribution and retail refers to the delivery of food items to the final consumers. In recent years, technological advances have significantly modified the organization of the retail supply chain, thus also influencing consumer behavior that is becoming more demanding regarding the quality and price of products delivered, time of delivery, and level of services offered. Nevertheless, certain technologies such as quick response codes, RFID and near-field communication networking, automated storage and retrieval systems, agent-based logistics, real-time monitoring using cameras, and delivery drone systems have already been implemented in retail supply [84]. The recent COVID-19 crisis indicated the existence of vulnerabilities, particularly concerning logistics and distribution causing unprecedented shocks to the food system [85]. Figure 6 shows various ICT tools used in food distribution and retail.

Although logistics and supply-chain optimization are new sectors for the utilization of artificial intelligence (AI) techniques, given the challenge of unforeseen disruptions and possible optimization, the use of the AI techniques is considered important as they help in enhancing the knowledge base of the field [86]. Toorajipour et al. [86] identified the most prevalent AI techniques in supply-chain management such as artificial neural networks (ANNs), fuzzy logic and models, multi-agent and agent-based systems, genetic algorithms (GAs), general forms of AI, data mining, and case-based reasoning. Machine-learning (ML) methods for near-infrared spectroscopy enabled a rapid, nondestructive, environmentally friendly, and accurate analysis of the composition, functional properties, and safety of a wide range of food products and raw materials, while the utilization of global ANN models has facilitated the international grain trade [87]. Min [88] emphasized the role of expert systems in solving complicated inventory control and planning problems in supply-chain management. Expert systems simulate human expertise in a particular field for decision-making activities. Their ability to capture dynamic complexity in the inventory patterns helps the inventory managers to estimate the appropriate levels of inventory without running short of stock as well as storing way beyond the requirements. An expert system can analyze huge databases of production schedules, bills of materials, order patterns, and other relevant data to determine the expected level of future orders and suitable timings for inventory replenishment. These expert systems are based on defining a set of rules that have already been proven to work. The numerous data collected during pandemics and other crises can be used to predict the extremes of hikes in demand and the extent of damages that happen in the supply chain with the help of expert systems. Similarly to this, the first indication of ML to determine the quantity of stored grain in model silos has recently been proposed by Duysak and Yigit [89]. The model was developed based on a KNN algorithm with an accuracy of 96.71%. Because grains are the most important, unperishable food sources for humans and animals, the amount of grain in the inventory must be known especially during crisis situations (e.g., war or famine).

If the emergency logistics demand during a crisis can be predicted, retail supply chains can be designed with more resistance to crises [90]. The emergency supply-chain demand is linked to the type of and also the severity of shock, as well as the location of its occurrence. The demand for emergency logistics needs to be dynamically determined considering all these factors. The system based on AI and machine learning (ML) to solve the dynamic demand forecasting during abnormal situations was proposed, which utilized a neighborhood-rough-set genetic algorithm—a support-vector-machine algorithm that is used to forecast the demand for emergency logistics. Their simulations have demonstrated that their model had significantly fewer errors than conventional SVM or neural network models. Ni et al. [91] emphasized the importance of using ML models to tackle the uncertainties and fluctuations in supply-chain management called the “bullwhip effect”. Machine language techniques are used to describe the nonlinear relationship among the factors to be considered in decision-making. For example, the sales of a particular beverage can be affected by the weather condition and governmental tax policy. Since their relationship is nonlinear, ML is the best solution in these scenarios. According to their review, grocery and food supply chains are the major beneficiaries of using ML techniques due to the necessity of making quick decisions and taking actions [91]. Although existing ICTs have introduced significant benefits to the food supply chain, it is important to securely and timely transfer the sensed data to the remotely located cloud server. Furthermore, the distribution and retail process highly depend on the flow of food, delivery time, and customer preferences requiring continuous monitoring. In addition, it is also necessary to conduct trend analysis of data gathered from stock management software and online food applications to predict the food consumption, customer demands, and food prices. This drives to exploring Industry 5.0 technologies such as blockchain and 6G that can support in the tracing and tracking of food and in securely and timely sharing the data. In addition, AI and DT enable food suppliers to conduct prediction analysis, virtually observe the food supply chain, and take smart decisions to improve productivity.

## 5. Key Enhancements to Food Supply Chain by Specific Industry 5.0 Technologies

The consequences of a crisis for a society depend on the level of society preparedness for the crisis, infrastructure in place, and training and skills of people engaged. The technologies (e.g., IoE, CC, blockchain, AI, DT, and 6G) of Industry 5.0 are yet to be fully explored and exploited in ensuring the resilience and sustainability of food systems. Therefore, this paper aimed to sketch out a complete concept of a food system using Industry 5.0 technologies, and the main focus has been given to the use of 6G, AI, blockchain, and digital twin (DT) at different processes from production to distribution and retail to help optimize the food supply chain and thwart any possible disruption thereof.

### 5.1. Internet of Everything, 6G, and AI 

**Figure 7:**
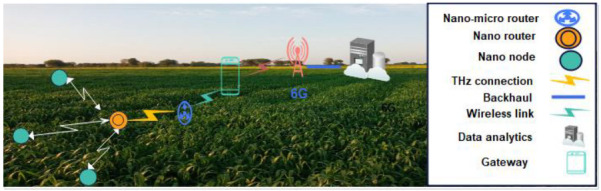
Envisioned smart production architecture using 6G.

The IoE extends the concept of the IoT; according to Bouzembrak et al. [92], when it comes to the Internet of Things (IoT) assisted applications, food supply chains top the chart. Monitoring of quality, safety, and traceability of the food products in collaboration with humans and supports of AI are the primary areas of concern that can be addressed by the IoE, especially when food is transported through longer supply chains. It is proposed to use the IoT to link the front-end activities of the food supply chain such as logistics, retail management, whole-sale operations, and warehouse management, which involve both humans and devices, to the back-end activities including food production, which is monitored using IoT tools. The integration of the IoT and humans with process- and data-driven new opportunities in the food supply chain arose. For instance, IoT sensors collect data such as soil, warehouse temperature, trace-and-track data from production, food processing and distribution, and retail units. Then, the gathered data are forwarded to a gateway and then to a remote server using wireless communication technologies e.g., WiFi, Bluetooth, LoRa, and 4G/5G. In the future, 6G is also expected to enhance the capabilities of existing communication technologies to achieve more reliable, fast, and larger connectivity that can help in increasing productivity in the industrial processes. For example, 6G enables the collection of data using nanonodes and the sending of the data to a nanorouter using terahertz (THz) communication; then the data are forwarded to a gateway using a nano–micro interface. The gateway is responsible for sending data over a 6G network following backhaul connectivity and then data analytics as given in Figure 7 [93].

Furthermore, technologies such as RFID technology, infrared (IR) sensors, global positioning systems (GPS), satellites, laser scanners, unmanned aerial vehicles (UAVs), cobots, and others enable timely delivery; locate the food items in transport; and monitor the quality of food during storage and transport to ensure that food-safety standards (HACCP, ISO 22000) are maintained, continuously monitor food conditions, and allow timely interventions to remove spoiled items before reaching the customer [94]. However, these devices collect a large quantity of data, which requires proper data processing to store and extract the important information. The rapid growth in cloud computing and AI applications motivates the utilization of these tools for data storage and predicting trends using historical data to enhance the supply management and decision-making process. The data analytics involves AI and ML such as computer vision, convolutional neural networks (CNNs), artificial neural networks (ANNs), fuzzy logic, neuro-fuzzy logic, expert systems, and other methodologies, which aim to feed machines with data from past experiences and statistical data, which enable the execution of the assigned task and solving a particular problem [95]. In addition, the huge data collection capabilities provided by the IoE will require new schemes to ensure trust and traceability in the food system.

The growing popularity of smart mobile phones and the advances in digital platforms have led to the development of e-commerce conducted via both traditional online websites and mobile apps [96]. In [97], mobile-based pork quality and safety tracing system has been developed to monitor the traceability of pork from pig breeding, purchase, sale, and slaughter services to table. In addition, digital technologies, e-commerce, and mobile apps are changing consumer experiences as well, enabling personalized offers in line with the diet, specific nutrition, or health needs of the consumer [98]. Food delivery applications have been widely adopted by catering businesses and customers, being extremely relevant during the recent COVID-19 crisis. Thus, the COVID-19 crisis has been turned into an opportunity to evolve social media into social marketing platforms allowing users direct contact with the food producers, choice of the payment mechanism, and pickup place. Zhao and Bacao [99] conducted a study to determine the users’ intention to use food delivery apps during the COVID-19 crisis and concluded that it has been determined by users’ satisfaction, perceived task-technology fitness, trust, performance expectancy, and social influence. The last mile delivery is the critical section of the supply-chain management. The threats to food security due to the COVID-19 crisis have been lively discussed lately [50]. Drones appeared to be a good alternative to support the delivery process, especially in terms of transportation cost, CO_2_ emissions, and congestion so that several big companies, such as Amazon, Google, UPS, DHL, and Domino’s Pizza, have started to use them for deliveries [100,101]. Food drone delivery in combination with mobile phone applications enables the scheduling and solving missed delivery problems, enables flexible delivery time, reduces the cost of transportation, and allows more sustainable delivery process [101]. Furthermore, blockchain has been suggested as a tool with a high potential to increase transparency, traceability, and sustainability in food supply chains, and hence decrease fraud and product falsification [46,102].

### 5.2. Trace and Track Using Blockchain Technology

The blockchain plays a key role in Industry 5.0 and is gaining significant attention in both the industry and academia [103]. The food supply chain involves different actors to ensure food safety and quality. However, food traceability also plays an important role in making the food supply chain more efficient. Thus, different track-and-trace technologies and software tools are used to ensure that the necessary checking and steps have been performed as required through all processes of production, postharvest operation and processing, and distribution and retail in the food system to improve food traceability. Traditionally, RFIDs and barcodes are the most common track-and-trace technologies in the food system.

**Figure 8:**
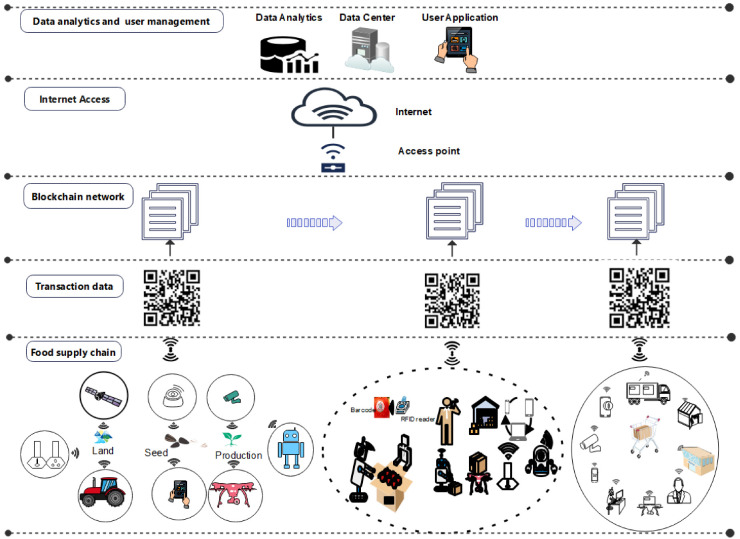
Blockchain in food supply chain.

The rapid growth of Industry 5.0 technologies drives the development of distributed trace-and-track systems that can avoid the risk of single-point failure in the system and can ensure integrity in the food supply chain. This motivates the use of blockchain technology that supports a robust information system and can help to improve traceability and transparency in the supply chain from production to distribution and retail. Figure 8 illustrates the proposed blockchain-based food supply chain, where data gathered from each process are shared and stored in a distributed ledger, which is immutable [64].

Blockchain technology verifies transactions via multiple nodes in the blockchain network; all transactions are secured, records of transactions are stored in blocks, and the blocks of data are stored in a chain, which create the blockchain [102], which employs smart contracts to automate the agreement process and allows the secure peer-to-peer exchange of data. Each block generates a hash code from its content, and it is referred to in the next block. Blockchain provides a mechanism in which every participant of the blockchain can verify if a block has been genuinely added or any unauthorized manipulation happened. It makes the data available in blockchain immutable and, thus, safe. It enables financial transactions among the untrusted parties without the requirement of intermediaries or centralized systems [104]. It has no intermediary or centralized authority to administer or control the transactions. The impact of blockchain technology has been examined by Kamilaris et al. [105] who indicated that blockchain can enable a transparent supply chain. The transparency and fault tolerance features of blockchain can solve problems in scenarios where numerous untrusted players participate [46]. Especially in food safety management in the agri-food supply chains, in accordance to food safety standards, the application of blockchain technology can ensure the trust among the participants [106,107], which is very critical in this domain. The common requirement during crisis is to backtrack the supply-chain operations to find out the source of the outbreak such as food contamination or any other factor that has gone against the regulation. Similarly, the systems that can identify those irregularities and make notifications to the authorities should be built [108]. Likewise, Kouhizadeh et al. [109] emphasized the technological, organizational, and environmental barriers for wider blockchain technology adoption for supply-chain management. Apart from that, several companies have started using blockchain in the food supply chain. The European retailer Carrefour is using blockchain to verify standards and trace food origins in various categories (e.g., meat, fish, fruits, vegetables, and dairy products) [110]. Walmart, Nestle, Bumble Bee foods, the Chinese e-commerce giant JD.com, and the Dutch supermarket chain Albert Heijn are the other big players that have also started using blockchain. Regarding traceability, the blockchain-based systems are more homogenous and enable interoperability among different entities involved in the food supply chain, as well as between diverse supply chains, bringing thus to a higher level the issues of traceability and abilities of food withdrawal from the market in accordance to food safety standards [111].

### 5.3. Digital Twin

Food processing involves different stages and actors from food production and storage to distribution and exploits low-cost sensors to monitor the food supply chain. This results in a significant amount of data being gathered, which brings new challenges on how to utilize the stored data in a more intelligent way to remotely optimize the production and supply-chain process. Nowadays, the digital twin is gaining strong consideration, which represents the virtual replica of a device or process including both of its elements and dimensions and how it works throughout its lifecycle in the virtual space [112]. DT is expected to enhance food production and to support in identifying failures and their causes during the supply chain, as well as to optimize decision-making while reducing food loss and maintenance time.

**Figure 9:**
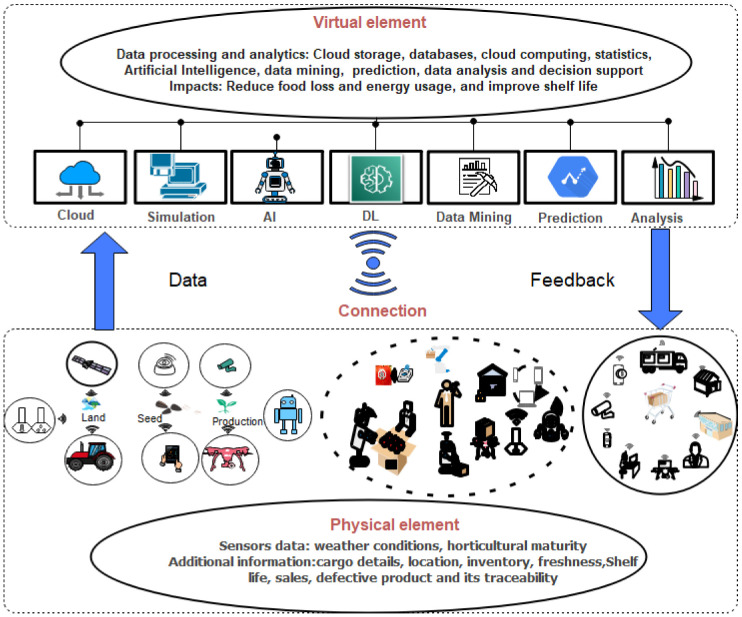
Digital twin for food supply chain.

A proposed digital twin for food supply chain consists of three main elements. First, the physical element consists of multiple sensors to measure different parameters from the production site or agriculture farm growing vegetables or fruits. These sensors collect data near the farm such as temperature, soil, climate, and irrigation conditions, as well as product freshness and horticultural maturity, and use available wireless links, e.g., WiFi and LoRaWAN, to transfer the data to the cloud for further analysis. In some scenarios, these sensors can also be mounted on other devices such as robots and drones to gather the data from the production field. Second, the virtual or digital element contains all necessary information about the physical product gathered from sensors (physical element) including weather conditions, product features, and its components, and the processes involved such as logistics and marketing, retailers, and consumers. Using the above details, DT will incorporate different data analytics, optimization approaches, and simulation platforms including software and AI techniques to identify and forecast potential problems in food processing that degrade food quality and induce food loss. Moreover, it will provide feedback to improve process performance and product quality, and actionable data, e.g., remaining shelf life, to facilitate the decision-making process. Finally, the connection is a foundation for the DT that provides the interaction of the physical object (i.e., deployed sensors) with the virtual object (i.e., cloud-based data storage). It plays a significant role by facilitating the data transfer collected using sensors and then accordingly updating the virtual state. Furthermore, it depends on different factors such as geographical conditions, the volume of data generated, data transmission rate, wireless technology, and delay requirements. Figure 9 proposes a general framework of DT in food processing from production to food consumers, which involves different steps, technologies, and techniques.

DT can give a better understanding of the interrelations involved in food processing by analyzing continuous and real-time data gathered using sensors and then implementing data analysis and optimization approaches to support actors in the decision-making process, predict future trends and financial losses, improve the user’s experience, and prevent the problems in the supply chain.

### 5.4. Cobots

Various technologies that feed into collaborative robots have been in existence, such as the IoT, robotics, augmented reality, and avatar. What is new is this concept of use of robots in a collaborative mode. It requires reimagining roles of both robots and humans. To begin with, one needs to accept that humans and robots are a hindrance to each other. That prepares the ground for leveraging strengths of each other—the creativity of human beings and accurate and fast repeatability of robots [20]. Lately, robots are no longer only large robots working from within safe cages. They are increasingly being built with small footprint, movability, and varying degrees of adaptability from one purpose to another. Therefore, one can imagine robots being deployed and redeployed with minimal planning and costs and help achieve critical goals of making a working environment safe, increasing productivity, and making profits. Thus, employees in various food sectors are now presented with a unique opportunity to work alongside robots—proving an adage that people should work with robots, not like robots. Imagine what it would mean to a person with disability to work as efficiently as an able person.

Today, the use of cobots in sustainable food production, storage, and distribution is being constructively reimagined, and this is an open academic research area drawing up ideas from Industry-5.0-driven smart manufacturing and logistics [113].

## 6. Conclusions

Information and communication technologies (ICTs) have transformed almost every industry in the recent past and currently represent one of the most vivid transformation processes in global agriculture and food systems. The recent COVID-19 pandemic and still an ongoing war crisis have indicated the vulnerability of the food system, which can be mitigated with the utilization of various technologies from the ICT domain depending on not only the nature and severity of crisis but also the specificity of the food supply chain. There are numerous ways of implementing these technologies, and they are continuously evolving. Apart from crisis situations, population growth and migrations, climate change, and changing consumer habits also necessitate major transformation to the food system in the future. Efficiency, sustainability, integrity, and resilience are going to be the primary objectives for innovations in food systems. Lately, with the emergence and evolution of Industry 5.0, there is an effort to use human expertise in collaboration with efficient, accurate, and intelligent machines to achieve resource-efficient and user-preferred manufacturing solution. Numerous promising technologies such as AI, big data analytics, digital twin, blockchain, cobots, 6G, Internet of everything (IOE), and edge or cloud computing are expected to assist Industry 5.0. Despite gaining significant attention in Industry 5.0, these technologies have limited consideration in food security, and this observation motivates us to provide a review on Industry 5.0 technologies and their implementation in each process of the food supply chain with expected benefits by exploiting the potential and opportunities of these technologies in each component of the food system. Thus, this paper summarized the possible use of emerging technologies of the industry today, namely Industry 5.0, in the food supply chain from production to distribution and retail. It highlights the benefits of key technologies such as big data analytics, AI, Internet of Everything (IoE), digital twin, blockchain, cobots, 6G, and edge or cloud computing that can be incorporated in food security and food system to achieve food sustainability by enabling food safety, quality, integrity, and security in the supply chain. The challenges and barriers in the implementation of these technologies such as Internet connectivity, security of devices and communication channels, storage requirements, government regulations, consumer acceptance, and, most importantly, costs must be evaluated. Despite the fact that the food sector represents one of the leading sectors in many economies around the globe, the implementation of technical solutions is still inadequate mainly due to not only the small margin of food items being unsupported toward larger investments, shortage of skilled staff, and availability of devices but also the food system non-uniformity around the world. The implementation of the innovative methods to ensure normal functioning of the food system should not result in loss or reduction of profits, and it must be carefully evaluated and the investments prioritized before applying any technology-based solution. Although some companies have launched different solutions for the management of their supply chains, the technical details about their implementation have not been reported. Moreover, in order to take the advantages provided by the technological advancements in the ICT domain, the modernization of food storage, processing, and distribution facilities must be carried out, together with the alleviation of the digital divide between different countries especially in long and more complex food systems.

## Data Availability

Not applicable.
